# Functioning, symptom expression and risk along the psychosis continuum

**DOI:** 10.1017/S0033291723001046

**Published:** 2023-11

**Authors:** Sarah Butter, Mark Shevlin, Orla McBride, Richard P. Bentall, Philip Hyland, Gerard Leavey, Jamie Murphy

**Affiliations:** 1 School of Psychology, Ulster University, Coleraine, Northern Ireland; 2 Bamford Centre for Mental Health & Wellbeing, Ulster University, Coleraine, Northern Ireland; 3 Department of Psychology, The University of Sheffield, Sheffield, England; 4 Department of Psychology, Maynooth University, Maynooth, Ireland

**Keywords:** Continuum, functioning, need for care, psychosis, risk factors, schizotypal

## Abstract

**Background:**

The psychosis continuum implies that subclinical psychotic experiences (PEs) can be differentiated from clinically relevant expressions since they are not accompanied by a ‘need for care’.

**Methods:**

Using data from Wave 2 of the National Epidemiologic Survey on Alcohol and Related Conditions (NESARC; *N* = 34 653), the current study examined variation in functioning, symptomology and aetiological risk across the psychosis phenotype [i.e. variation from (i) no PEs, ‘No PEs’ to (ii) non-distressing PEs, ‘PE-Experienced Only’ to (iii) distressing PEs, ‘PE-Impaired’ to (iv) clinically defined psychotic disorder, ‘Diagnosed’].

**Results:**

A graded trend was present such that, compared to those with no PEs, the Diagnosed group had the poorest functioning, followed by the PE-Impaired then PE-Experienced Only groups. In relation to symptom expression, the PE-Impaired group were more likely than the PE-Experienced Only and the Diagnosed groups to endorse most PEs. Predictors of group membership tended to vary quantitatively rather than qualitatively. Trauma, current mental health diagnoses (anxiety and depression) and drug use variables differentiated between all levels of the continuum, with the exception of the extreme end (PE-Impaired *v*. Diagnosed). Only a few variables distinguished groups at the upper end of the continuum: female sex, older age, unemployment, parental mental health hospitalisation and lower likelihood of having experienced physical assault.

**Conclusions:**

The findings highlight the importance of continuum-based interpretations of the psychosis phenotype and afford valuable opportunities to consider if and how impairment, symptom expression and risk change along the continuum.

## Introduction

Psychotic experiences (PEs) are reported by many ‘healthy’ individuals who are not diagnosed with a psychotic disorder (Baumeister, Sedgwick, Howes, & Peters, 2017; McGrath et al., 2015; van Os, Linscott, Myin-Germeys, Delespaul, & Krabbendam, 2009). The well-documented psychosis continuum posits that this is a result of an extended psychosis phenotype (Linscott & van Os, 2013; van Os et al., 2009). According to this hypothesis, subclinical PEs should be differentiated from clinically relevant expressions of psychosis by considering an individual's ‘need for care’.

It has been reported that non-clinical individuals (those with enduring PEs but no diagnosis or need for treatment), despite experiencing hallucinations and other first-rank symptoms (Brett, Peters, & McGuire, 2015; Peters et al., 2016), have fewer negative symptoms, cognitive difficulties and attentional anomalies compared to ultra-high-risk help-seeking and clinically diagnosed groups (Brett et al., 2015; Peters et al., 2016). van Nierop et al. (2012) reported that, compared to a control group, individuals with self-reported PEs which were not confirmed by a clinical interview (‘false-positive’ group) had higher rates of mood, anxiety and substance use disorders, trauma and negative life events, and poorer physical, mental and social functioning. When compared to those with clinically confirmed PEs (‘true-positive’ group), however, these associations were generally smaller. Additionally, individuals who experience non-clinical PEs are significantly more likely than those without PEs to engage in help-seeking behaviour (e.g. see GP for emotional problems, attend counselling) (DeVylder, Oh, Corcoran, & Lukens, 2014; Murphy, Shevlin, Houston, & Adamson, 2012). Furthermore, greater perceived control, less distress, more positive (e.g. spiritual) appraisals and normalising responses have generally been reported to distinguish non-clinical from diagnosed groups (Bak et al., 2003; Baumeister et al., 2017; Brett et al., 2007; Brett, Heriot-Maitland, McGuire, & Peters, 2014; Johns et al., 2014; Powers, Kelley, & Corlett, 2017).

As yet, little research has considered variation in functioning, symptom expression and aetiological risk at levels that encapsulate and correspond to a broader description and conceptualisation of the psychosis phenotype [i.e. variation from (i) no PEs to (ii) non-distressing PEs to (iii) distressing PEs to (iv) clinically defined psychotic disorder]. This is surprising given how informative evidence of variation at each of these levels could be; determining if functional impairment, symptomology and risk factors vary at each of these ‘levels’ may help us to understand (a) whether impaired functioning is associated with psychosis experience at all levels of the phenotype or whether it is something that is specifically reflective of distressing and clinically captured experiences only, (b) whether the defining characteristics of the phenotype (symptom expression) are stable or variable along the continuum, and if so, how and where, and (c) what factors are responsible for transitions along the continuum and where. In line with previous research on the psychosis continuum and need for care, it was broadly hypothesised that there would be similarities in areas of impaired functioning, symptom expression and risk factors along the continuum. However, it was expected that functioning would be poorer, PEs would be more likely to be endorsed and risk factors would be more strongly associated with psychosis expression moving from the lower to the upper end of the continuum.

## Method

### Sample

The National Epidemiologic Survey on Alcohol and Related Conditions (NESARC) is a longitudinal survey designed to be representative of the civilian, non-institutionalised adult population of the USA, including the residents of the District of Columbia, Alaska and Hawaii (Grant & Kaplan, 2005; Grant, Kaplan, Shepard, & Moore, 2003b). Respondents included those living in private households, military personnel living off base and people residing in non-institutionalised group housing. One adult was randomly selected from each dwelling (Grant & Dawson, 2006).

Wave 1 was conducted between 2001 and 2002. Face-to-face computer-assisted personal interviews (CAPI) were conducted by trained laypersons on 43 093 adults (81.0% response rate; Grant et al., 2003b). At Wave 2 (2004–2005), 34 653 eligible respondents were re-interviewed (86.7% response rate). The cumulative response rate for both waves combined was 70.2% (Grant & Kaplan, 2005). At both waves, data were weighted, clustered on primary sampling units and stratified to be representative of the US general population on a range of sociodemographic variables, based on the 2000 Decennial Census (Grant & Dawson, 2006). Descriptions of the survey design and data collection processes are available in greater detail elsewhere (Grant et al., 2003b; Grant & Kaplan, 2005).

### Measures

The Alcohol Use Disorder and Associated Disabilities Interview Schedule – DSM-IV version (AUDADIS-IV; Grant & Dawson, 2000) is a fully structured, self-report, diagnostic CAPI designed to be administered by clinicians or trained laypersons (Grant & Dawson, 2000). It assesses both past year and lifetime occurrence of a variety of psychiatric disorders, including personality disorders (Grant et al., 2003a). The AUDADIS-IV measures of psychiatric disorders have been shown to demonstrate high reliability in general population samples (Grant et al., 2003a; Ruan et al., 2008).

#### PEs

Sixteen PEs were drawn from the ‘unusual feelings and actions’ section of the AUDADIS-IV at Wave 2, each of which mapped onto one of three distinct schizotypal personality dimensions; ‘social/interpersonal’, ‘disorganisation’ and ‘cognitive/perceptual’ (see Table 2). Respondents were asked if they had ever experienced each PE (i.e. ‘Most of the time throughout your life, regardless of the situation or whom you were with…’, Yes/No response option). If yes, a follow-up item enquired if any distress or impaired functioning had been associated with that PE [i.e. ‘Did this (experience) ever trouble you or cause problems at work or school, or with your family or other people?’]. Additionally, respondents were asked at Wave 1 if they had ever been diagnosed with schizophrenia or a psychotic disorder/episode by a doctor or other health professional. At Wave 2, respondents were asked if they had been diagnosed since their Wave 1 interview. Information from both waves was used to categorise lifetime psychotic disorder/episode.

#### PE groups

**Figure 1.**
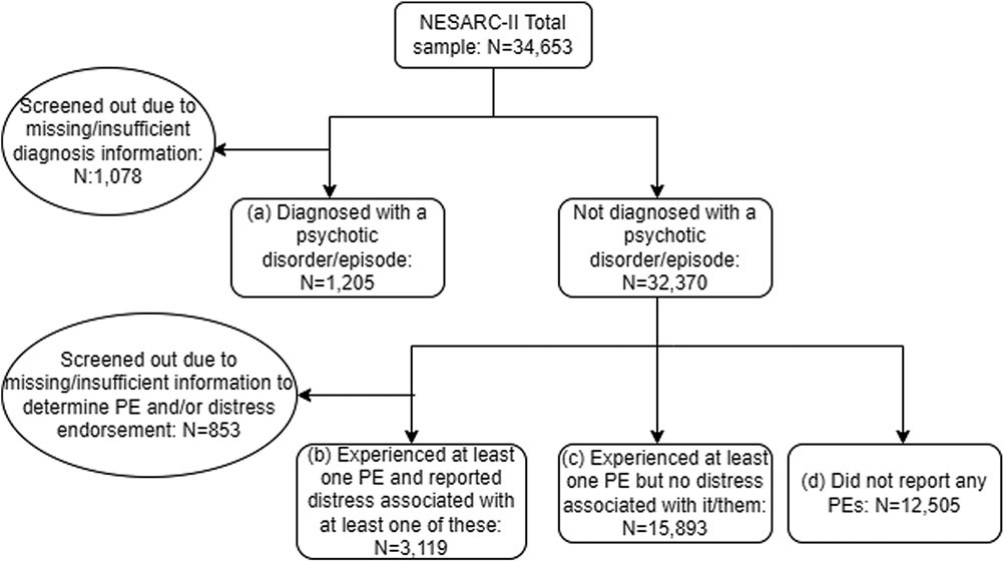
Flow diagram detailing categorisation into PE groups: (a) Diagnosed, (b) PE-Impaired, (c) PE-Experienced Only and (d) No PEs.

Based on responses to the abovementioned PE items, individuals were categorised into four groups (unweighted *N*, weighted %; see Fig. 1):

- Diagnosed with a psychotic disorder/episode [*N* = 1205 (3.1%); ‘Diagnosed’]: This group comprised individuals who reported a lifetime diagnosis of schizophrenia or another psychotic disorder or episode. Inclusion in this category was made regardless of respondents' endorsement of PE items.
- PEs with distress or impairment [*N* = 3119 (8.6%); ‘PE-Impaired’]: This group comprised individuals who endorsed at least one of the 16 PE items and reported distress/impairment associated with at least one of these experience(s). However, these individuals did not report being diagnosed with a psychotic disorder/episode.
- PEs without distress or impairment [*N* = 15 893 (45.4%); ‘PE-Experienced Only’]: This group comprised individuals who had endorsed at least one of the 16 PE items but did not report any distress/impairment associated with any of these experiences, nor had they reported being diagnosed with a psychotic disorder/episode.
- No psychotic disorder diagnosis nor endorsement of PEs [*N* = 12 505 (37.6%); ‘No PEs’]: This group comprised individuals who neither reported a lifetime diagnosis of psychotic disorder/episode nor any of the 16 PEs.

Individuals who could not be categorised into one of these four groups due to missing data were removed from the analysis [*N* = 1931 (5.2%)].

#### Functioning

Impaired functioning was measured at Wave 2 using the Short-Form 12 Health Survey (SF-12v2; Ware, Kosinski, Turner-Bowker, & Gandek, 2002), a 12-item measure of current functioning and life satisfaction over the past 4 weeks. The SF-12v2 produces norm-based scores across eight subscales: physical functioning, role physical functioning (i.e. how physical health interferes with regular activities), bodily pain, general health, vitality, social functioning, role emotional functioning (i.e. how emotional health interferes with regular activities) and mental health. Scores are standardised and range from 0 to 100 (M = 50, s.d. *=* 10). Higher scores reflect better functioning (Ware et al., 2002). The SF-12v2 has been reported as a reliable and valid measure in both general population samples (Kim et al., 2014; Montazeri et al., 2011) and in those with serious mental health or behavioural difficulties (Huo, Guo, Shenkman, & Muller, 2018).

#### Predictor variables

Using the NESARC Wave 2 variables, an attempt was made to match the sociodemographic, environmental and psychological variables used by Peters et al. (2016). The following were used as predictor variables in the analysis:

- *Sex:* Male (1), female (0).
- *Age:* Six age categories ranging from 24 years old or younger (0) through to being 65 years old or older (5).
- *Children:* Has no children (1) or at least one child (0).
- *Ethnicity:* Ethnic background was recoded into a dichotomous variable, which identified respondents as either White (0) or other ethnicity (1).
- *Relationship status:* Identified respondents as either being married or living with their partner (0) or not married/living with partner (1).
- *Education:* Has completed high school education (0) or not (1).
- *Unemployment:* Unemployed (1) or not unemployed (0; i.e. employed, student, retired, etc.).
- *Parental mental health:* This indicated whether, before age 18, the respondent had a parent/other adult living in the home who had been treated or hospitalised for mental illness (1) or not (0).
- *Religious services:* Current attendant of places of worship (1) or not (0).
- *Importance of religion:* Respondents were asked whether religious or spiritual beliefs were important in their daily lives. This was recoded into a binary variable whereby responses of ‘very important’ were coded as 1 and all other responses (somewhat important, not very important and not important at all) were coded as 0.
- *Migrant:* Identified respondents' parents' country of origin either as outside the USA (1) or at least one parent being from the USA (0).
- *Cannabis use:* Information from the ‘medicine use’ section of the questionnaire was used to identify if respondents had used cannabis since their last interview at Wave 1 (yes = 1, no = 0).
- *Other drug use:* Similarly, a separate variable was created to identify if respondents had used any other drug (sedatives, tranquilisers, opioids, amphetamines, cocaine, hallucinogens, inhalants, heroin, other) since their last interview (yes = 1, no = 0).
- *Sexual assault:* During the ‘traumatic events’ section of the questionnaire, respondents were asked if they were ever sexually assaulted, molested or raped or if they ever experience unwanted sexual activity (1) or not (0).
- *Physical assault:* A binary variable was created which indicated whether respondents had ever been physically attacked or badly beaten or injured by their parents, their partner or someone else (1) or not (0).
- *Mugged:* Respondents were asked if they had ever been mugged, held up or threatened with a weapon (1) or not (0).
- *Parental neglect:* This variable indicated whether respondents had been seriously neglected by either of their parents/guardians before the age of 18 (1) or not (0).
- *Family bonding:* Five items from the ‘background information’ section of the questionnaire enquired about family bonding and support. Responses were scored on a five-point Likert scale ranging from ‘never true’ to ‘very often true’. Responses were recoded into a binary variable whereby ‘never true’ or ‘rarely true’ indicated a lack of family support (1) and all other responses (sometimes true, often true or very often true) indicated family support (0).
- *Major depressive* (*MD*) *episode:* Respondents who met the diagnostic criteria for major depressive episode within the past 12 months (excluding substance-induced disorders or those due to a medical condition) were categorised yes (1) and no (0).
- *Generalised anxiety disorder* (*GAD*)*:* Respondents who met the diagnostic criteria for GAD over the past 12 months (excluding substance-induced disorders or those due to a medical condition) were categorised yes (1) and no (0).

For all variables, ‘unknown’ responses were treated as missing data.

### Analytic plan

Firstly, multivariate multiple regression analysis was conducted to assess whether degree of functional impairment differed across the PE groups. This analysis allows several dependent variables (the eight SF-12v2 subscales) to be jointly regressed on all predictor variables. The predictor variables in this model included dummy coded PE group variables (Diagnosed, PE-Impaired and PE-Experienced Only, with the No PE group as the reference category) and control variables (age, sex). The estimated regression coefficient for each variable indicates the mean difference between the specific PE category and the No PE group, and the associated confidence intervals (CIs) were used to indicate statistical significance. This approach was used as (1) all the model parameters are estimated simultaneously thereby avoiding the need for post-hoc adjustment for multiple testing, (2) the use of robust maximum likelihood (MLR) estimation allows for missing data to be handled efficiently by using all available data (Schafer & Graham, 2002), and third, MLR estimation is robust against deviations from normality and produces unbiased standard errors (West, Finch, & Curran, 1995). Next, the proportion of respondents endorsing PEs, across the full sample and within the Diagnosed, PE-Impaired and PE-Experienced Only groups, was examined and χ^2^ tests of association with pairwise *z*-tests (Bonferroni adjusted) were conducted to compare proportions across all groups. Finally, three separate multinomial logistic regressions were conducted to assess whether sociodemographic, substance use, trauma, family bonding and diagnostic predictor variables could discriminate between PE group membership. In each analysis, a different reference category was used to ensure that all PE group comparisons were estimated. Analyses were carried out in SPSS v27 and Mplus 8.3 (Muthén & Muthén, 2017) using survey design variables.

## Results

Sample characteristics are presented in online Supplementary Table S1.

### Functioning

Table 1 reports the unstandardised regression coefficients and CIs for the Diagnosed, PE-Impaired and PE-Experienced Only groups (with No PEs as the reference category) along with age and sex, predicting the total score on each of the SF-12v2 subscales. A graded trend was present across all eight SF-12v2 subscales, such that compared to the No PEs group, the Diagnosed group had the poorest functioning, followed by the PE-Impaired then PE-Experienced Only groups.

**Table 1.** Unstandardised (*B*) regression coefficients for SF-12v2 subscales (*N* = 32 714)^a^

	Physical	Role physical	Bodily pain	General health	Vitality	Social	Role emotional	Mental health
Predictors	*B* (95% CI)	*B* (95% CI)	*B* (95% CI)	*B* (95% CI)	*B* (95% CI)	*B* (95% CI)	*B* (95% CI)	*B* (95% CI)
Age	−2.19 (−2.27 to −2.11)	−1.74 (−1.82 to −1.65)	−1.52 (−1.60 to −1.44)	−2.04 (−2.14 to −1.94)	−0.89 (−0.97 to −0.81)	−0.60 (−0.69 to −0.51)	−0.86 (−0.95 to −0.77)	0.14 (0.06–0.23)
Sex (male)	1.73 (1.47–1.99)	1.51 (1.26–1.75)	1.34 (1.08–1.60)	0.58 (0.29–0.88)	2.48 (2.19–2.76)	1.53 (1.31–1.75)	1.77 (1.50–2.04)	2.41 (2.12–2.71)
Diagnosed	−5.90 (−7.02 to −4.78)	−6.71 (−7.64 to −5.77)	−7.94 (−8.99 to −6.88)	−8.33 (−9.50 to −7.16)	−6.11 (−7.01 to −5.21)	−8.36 (−9.42 to −7.30)	−8.91 (−10.00 to −7.82)	−8.35 (−9.35 to −7.35)
PE-Impaired	−2.86 (−3.33 to **−**2.38)	−3.87 (−4.34 to −3.39)	−4.99 (−5.57 to −4.42)	−4.40 (−4.97 to −3.83)	−4.82 (−5.32 to −4.32)	−6.21 (−6.78 to −5.65)	−5.80 (−6.31 to −5.28)	−7.36 (−7.88 to −6.84)
PE-Experienced Only	−0.93 (−1.21 to **−**0.66)	−1.25 (−1.53 to −0.96)	−1.80 (−2.10 to −1.51)	−1.74 (−2.05 to −1.42)	−1.49 (−1.78 to −1.20)	−1.32 (−1.56 to −1.08)	−1.32 (−1.61 to −1.03)	−1.91 (−2.19 to −1.63)
*R*^2^	0.13	0.10	0.08	0.10	0.05	0.06	0.06	0.07

a Weighted, clustered and stratified using survey design variables.

### PE prevalence and symptom expression

Overall, 60% of the total sample reported having experienced at least one of the 16 PE items (Table 2). Social/interpersonal PEs were the most commonly endorsed, with almost half the total sample reporting at least one of these experiences. More than a quarter had experienced at least one cognitive/perceptual PE, while less than a fifth reported any disorganised PE. This trend was also present across PE groups. The χ^2^ tests of association were significant for all 16 PEs, indicating that there was an association between PE endorsement and PE group. Overall, the PE-Impaired group was more likely than the PE-Experienced Only group to endorse all 16 PEs and was also more likely than the Diagnosed group to endorse 15 of the 16 PEs. Moreover, the Diagnosed group was more likely than the PE-Experienced Only group to endorse 13 of the PEs.

**Table 2.** PE item endorsement across total sample and PE groups*

		*N* (%)				
Item	Label	Total NESARC-II sample 34 653 (100.0)	PE-Experienced Only 15 893 (45.4)	PE-Impaired 3119 (8.6)	Diagnosed 1205 (3.1)	χ^2^, df, *p*
Social/interpersonal						
Have you had trouble expressing your emotions and feelings?	Express	4694 (13.5)	2359 (15.3)^a^	1753 (58.8)^b^	283 (24.3)^c^	2800.06 (1.82), *p* < 0.001
Have you rarely shown emotion?	Emotion	5720 (16.2)	3929 (25.0)^a^	1185 (38.6)^b^	275 (21.6)^c^	255.68 (2.0) *p* < 0.001
Often you felt nervous when with other people even whom you have known for a while?	Nervous	2253 (6.0)	1053 (6.4)^a^	818 (25.7)^b^	230 (18.5)^c^	1147.09 (1.89), *p* < 0.001
Have you felt suspicious of people, even if you have known them for a while?	Suspicious	4412 (11.0)	2490 (13.8)^a^	1324 (39.8)^b^	315 (23.8)^c^	1177.37 (1.96), *p* < 0.001
Have you often had the feeling of being watched or stared at, when around people?	Watched	3330 (8.3)	1839 (10.2)^a^	1040 (30.6)^b^	252 (20.1)^c^	929.92 (1.98), *p* < 0.001
Have there been very few people that you're really close to outside of your immediate family?	Close to	11 130 (30.4)	8093 (49.6)^a^	1830 (57.6)^b^	556 (43.7)^c^	87.47 (1.91), *p* < 0.001
Any social/interpersonal		17 008 (47.6)	12 388 (76.9)^a^	2976 (95.6)^b^	773 (63.9)^c^	694.94 (1.97), *p* < 0.001
Disorganised						
Have people thought you acted strangely?	Act strange	2810 (7.8)	1447 (9.5)^a^	897 (27.8)^b^	258 (22.3)^c^	984.90 (1.78), *p* < 0.001
Have people thought you have strange ideas?	Strange ideas	4343 (12.2)	2642 (17.0)^a^	1140 (36.6)^b^	268 (22.9)^c^	609.24 (1.81), *p* < 0.001
Have people thought you are odd, eccentric or strange?	Odd	3658 (10.0)	2084 (13.2)^a^	1030 (31.9)^b^	281 (23.6)^c^	685.98 (1.96), *p* < 0.001
Any disorganised		6178 (17.4)	3831 (24.7)^a^	1560 (49.0)^b^	402 (34.2)^c^	753.08 (1.93), *p* < 0.001
Cognitive/perceptual						
Have you had personal experiences with the supernatural?	Supernatural	3098 (8.4)	2017 (12.6)^a^	677 (20.9)^b^	185 (15.4)^c^	150.09 (1.94), *p* < 0.001
Have you had the sense that some force is around you, even though you cannot see anyone?	Force	6454 (18.0)	4502 (28.3)^a^	1234 (39.4)^b^	309 (25.9)^c^	157.36 (1.95), *p* < 0.001
Have you believed that you have a ‘sixth sense’ that allows you to know and predict things that others can't?	Sixth	3192 (7.8)	2047 (11.1)^a^	717 (21.4)^b^	191 (14.7)^c^	241.74 (1.97), *p* < 0.001
Have you often seen auras or energy field around people?	Auras †	963 (2.3)	548 (3.0)^a^	271 (7.3)^b^	78 (7.3)^c^	162.89 (1.95), *p* < 0.001
Have you ever felt you could make things happen just by making a wish or thinking?	Happen	2462 (6.3)	1562 (9.1)^a^	577 (16.4)^b^	151 (14.0)^c^	160.24 (1.97), *p* < 0.001
Have you often had the feeling that things that have no special meaning to most people are really meant to give you a message?	Meaning	3348 (8.4)	2063 (11.5)^a^	876 (25.5)^b^	217 (17.0)^c^	427.91 (1.76), *p* < 0.001
Have you often thought that objects or shadows are really people or animals, or that noises are actually people's voices?	Shadows	608 (1.5)	271 (1.6)^a^	197 (5.5)^b^	88 (7.1)^c^	273.99 (1.94), *p* < 0.001
Any cognitive/perceptual		10 640 (29.2)	7670 (46.5)^a^	1940 (60.5)^b^	524 (44.5)^c^	208.17 (1.95), *p* < 0.001
No. PE items						
0		12 797 (40.5)	0 (0.0)^a^	0 (0.0)^a^	292 (24.1)^b^	7720.74 (8.19), *p* < 0.001
1		7347 (21.9)	6528 (42.9)^a^	247 (8.1)^b^	210 (17.5)^c^	
2		4679 (13.5)	3843 (24.2)^a^	443 (15.0)^b^	152 (15.0)^b^	
3		2926 (7.9)	2165 (13.1)^a^	455 (14.8)^b^	129 (9.6)^c^	
4		2024 (5.5)	1350 (8.2)^a^	474 (15.6)^b^	77 (5.1)^c^	
5 or more		4168 (10.7)	2007 (11.6)^a^	1500 (46.5)^b^	345 (28.6)^c^	

*Unweighted *N*, weighted using survey design variables %; χ^2^ based on weighted, clustered and stratified data; ^abc^group comparisons based on weighted only data; differing subscript letters within a row denote item proportions differ significantly from each other at the 0.05 level (Bonferroni adjusted); data missing across PE items 0.6–1.3%; row and column *N*s may not total 100% due to missing data.

† Despite the same proportions reported for the Diagnosed and PE-Impaired groups, analysis indicated a statistically significant difference between these groups. We believe that this unusual finding may be as a result of the process of weighting the data.

Regarding endorsement of PE items at the distress/impairment level (see online Supplementary Table S2), χ^2^ tests revealed that there was a significant association between PE group (i.e. PE-Impaired or Diagnosed) and 14 of the 16 PE items. The PE-Impaired group had a significantly higher proportion of distress endorsement than the Diagnosed group in all of these cases, with the exception of ‘Shadows’. Regarding item count, 77% of the Diagnosed group reported not being impaired by any of the PEs (this included missing responses). A significantly higher proportion of the PE-Impaired group reported being distressed by one (56.1% *v.* 7.6%), two (21.8% *v.* 4.9%), three (9.8% *v.* 2.3%), four (5.2% *v.* 2.7%) and five or more PEs (7.0% *v.* 5.9%).

### Predicting PE group membership

Table 3 presents the results of the multinomial logistic regression analyses comparing predictor variables at different points along the psychosis continuum. In general, when compared to the No PEs reference group (columns 2–4), when risk was present, it was present across all the PE groups. Having any PE experience (PE-Experienced Only, PE-Impaired or Diagnosed) was associated with being male, non-White ethnicity, being unemployed, not being married/cohabiting with partner, having a parent/other adult living in the home being hospitalised due to their mental health before age 18, recent cannabis and other drug use, experiencing sexual assault, physical assault, being mugged and parental neglect, and past year MD episode and GAD. While these same variables generally tended to differentiate the Diagnosed and PE-Impaired groups from the PE-Experienced Only group (columns 5 and 6), the sociodemographic factors of non-White ethnicity, unemployment, not cohabiting with partner and parent mental health hospitalisation were specific to the Diagnosed group.

**Table 3.** Multinomial logistic regression analyses predicting PE group membership (*N* = 32 027)^†^

	OR (95% CI)			OR (95% CI)		OR (95% CI)
	Column 2	Column 3	Column 4	Column 5	Column 6	Column 7
Predictors	Diagnosed^a^	PE-Impaired^a^	PE-Experienced Only^a^	Diagnosed^b^	PE-Impaired^b^	Diagnosed^c^
Sociodemography						
Male	1.26 (1.04–1.53)*	1.70 (1.50–1.93)***	1.33 (1.24–1.42)***	0.95 (0.79–1.15)	1.28 (1.14–1.45)***	0.74 (0.60–0.92)**
Age						
<24	0.30 (0.19–0.48)***	1.45 (1.11–1.89)**	0.93 (0.81–1.08)	0.32 (0.21–0.50)***	1.55 (1.21–1.98)***	0.21 (0.13–0.33)***
25–34	0.37 (0.27–0.49)***	1.42 (1.16 –1.73)***	0.85 (0.77–0.94)**	0.43 (0.32–0.57)***	1.67 (1.38–2.02)***	0.26 (0.19–0.35)***
35–44	0.42 (0.33–0.55)***	1.30 (1.08 –1.57)**	0.82 (0.75–0.90)***	0.51 (0.40–0.66)***	1.58 (1.32–1.90)***	0.32 (0.25–0.43)***
35–44	0.72 (0.58–0.89)**	1.44 (1.20–1.73)***	0.97 (0.88–1.07)	0.74 (0.60–0.91)**	1.49 (1.24–1.78)***	0.50 (0.38–0.64)***
55–64	0.81 (0.63–1.05)	1.36 (1.12–1.65)**	0.98 (0.88–1.08)	0.83 (0.66–1.05)	1.40 (1.15–1.69)***	0.60 (0.46–0.77)***
Other ethnicity	1.63 (1.34–1.98)***	1.34 (1.19–1.51)***	1.35 (1.26–1.46)***	1.21 (1.00–1.46)*	0.99 (0.88–1.12)	1.22 (0.99–1.50)
No children	1.16 (0.94–1.42)	1.01 (0.89–1.15)	1.00 (0.93–1.09)	1.15 (0.95–1.41)	1.01 (0.90–1.14)	1.14 (0.92–1.42)
Unemployed	3.10 (2.53–3.79)***	1.22 (1.03–1.43)*	1.15 (1.02–1.30)**	2.68 (2.20–3.27)***	1.05 (0.90–1.23)	2.55 (2.03–3.20)***
<High school qualification	1.07 (0.85–1.34)	0.98 (0.84–1.15)	0.94 (0.85–1.03)	1.14 (0.91–1.44)	1.05 (0.89–1.23)	1.09 (0.83–1.43)
Not married/living with partner	1.46 (1.26–1.69)***	1.25 (1.12–1.39)***	1.19 (1.12–1.26)***	1.23 (1.06–1.42)**	1.05 (0.95–1.17)	1.17 (0.98–1.39)
Attend religious service	0.85 (0.72–1.02)	0.82 (0.73–0.93)**	0.87 (0.80–0.93)***	0.99 (0.83–1.16)	0.95 (0.84–1.07)	1.04 (0.86–1.26)
Religious/spiritual beliefs important	1.16 (0.96–1.41)	1.14 (0.99–1.31)	1.22 (1.13–1.33)***	0.95 (0.78–1.15)	0.93 (0.82–1.05)	1.02 (0.82–1.28)
Parent mental health hospitalisation	2.51 (1.88–3.35)***	1.41 (1.11–1.80)**	1.32 (1.13–1.53)***	1.91 (1.45–2.51)***	1.07 (0.87–1.33)	1.78 (1.33–2.38)***
Both parents born outside USA	0.89 (0.73–1.08)	0.71 (0.59–0.85)***	0.77 (0.71–0.83)***	1.16 (0.95–1.41)	0.93 (0.78–1.10)	1.25 (0.95–1.65)
Substance use						
Cannabis SLI	1.66 (1.18–2.32)**	1.55 (1.24–1.93)***	1.37 (1.17–1.62)***	1.21 (0.86–1.69)	1.13 (0.92–1.38)	1.07 (0.76–1.50)
Other drugs SLI	1.71 (1.26–2.33)***	2.03 (1.65–2.50)***	1.24 (1.04–1.49)*	1.38 (1.02–1.88)*	1.64 (1.39–1.93)***	0.84 (0.62–1.15)
Traumas						
Sexual assault	2.30 (1.76–3.02)***	2.37 (1.96–2.85)***	1.57 (1.38–1.79)***	1.46 (1.15–1.87)**	1.50 (1.28–1.76)***	0.97 (0.74–1.29)
Physical assault	1.89 (1.49–2.41)***	2.55 (2.19–2.96)***	1.66 (1.48–1.85)***	1.14 (0.89–1.47)	1.54 (1.35–1.75)***	0.74 (0.57–0.97)*
Mugged/threatened with weapon	1.95 (1.58–2.39)***	2.00 (1.71–2.33)***	1.56 (1.40–1.72)***	1.25 (1.00–1.56)*	1.28 (1.11–1.48)***	0.98 (0.78–1.23)
Parental neglect	3.00 (1.99–4.52)***	2.34 (1.72–3.19)***	1.64 (1.29–2.09)***	1.83 (1.25–2.67)**	1.43 (1.12–1.82)**	1.28 (0.87–1.88)
Family bonding (never or rarely)						
Family wanted me to be a success	1.08 (0.79–1.48)	0.81 (0.64–1.02)	0.87 (0.76–0.99)*	1.25 (0.93–1.69)	0.93 (0.75–1.15)	1.34 (0.96–1.87)
Family helped me feel like I was important or special	1.22 (0.86–1.75)	1.10 (0.83–1.48)	1.10 (0.92–1.33)	1.11 (0.79–1.56)	1.00 (0.76–1.33)	1.11 (0.73–1.68)
Family was a source of strength and support	0.94 (0.63–1.39)	0.99 (0.73–1.35)	1.09 (0.89–1.34)	0.86 (0.59–1.24)	0.90 (0.69 –1.19)	0.95 (0.62–1.45)
Part of a close-knit family	1.06 (0.72–1.57)	1.57 (1.22–2.03)***	1.19 (1.01–1.40)*	0.90 (0.62–1.29)	1.32 (1.05–1.67)*	0.68 (0.44–1.03)
Family believed in me	1.09 (0.69–1.72)	1.14 (0.79–1.64)	0.8 (0.68–1.07)	1.28 (0.83–1.98)	1.33 (0.96–1.85)	0.96 (0.59–1.57)
Psychiatric diagnoses						
Major depressive episode PY	3.86 (3.01–4.97)***	4.65 (3.91–5.53)***	1.55 (1.36–1.77)***	2.49 (1.95–3.18)***	3.00 (2.56–3.51)***	0.83 (0.64–1.09)
Generalised anxiety disorder PY	3.99 (2.93–5.44)***	4.70 (3.57–6.19)***	2.40 (1.93–2.99)***	1.66 (1.25–2.21)***	1.96 (1.61–2.38)***	0.85 (0.63–1.16)

PY, past year; SLI, since last interview.

^†^Weighted, clustered and stratified using survey design variables; *p < 0.05, **p < 0.01, ***p < 0.001. Reference categories: ^a^No PEs, ^b^PE-Experienced Only, ^c^PE-Impaired. Significant ORs in bold.

Finally, at the upper end of the continuum (column 7), few variables differentiated the Diagnosed and PE-Impaired groups. The Diagnosed group were more likely to be female, older, unemployed, have had a parent hospitalised due to their mental health, and less likely to have experienced physical assault. Overall, ORs varied; in some cases, a clear graded pattern was present such that the highest ORs were associated with the Diagnosed group, reducing for the PE-Impaired group and again for the PE-Experienced Only group (e.g. parental neglect, cannabis use, unemployment). However, in several cases, the PE-Impaired group were at increased risk compared to the Diagnosed group (e.g. other drug use, physical assault, and MD and GAD diagnoses). An overall trend was also present for age in that PE-Impaired group were the youngest while the Diagnosed group were the oldest.

## Discussion

The purpose of this study was to explore functional impairment, symptom expression and aetiological risk across the psychosis continuum.

### Functioning

Two main findings are noted in relation to functioning. Firstly, as expected, the findings of the current study suggest that individuals who report no distress/impairment associated with their PEs still experience compromised functioning across all domains, compared to individuals who have not experienced PEs. Thus, despite not having a need for care, these individuals experience physical, emotional and social impairments. Age and sex were controlled for in this analysis, therefore, it is possible that these individuals' functional impairments are the result of other factors, such as comorbid emotional or behavioural issues rather than a direct result of their PEs (DeVylder et al., 2014; Murphy et al., 2012), or that these deficits in functioning (e.g. poor physical health, reduced mobility, social isolation) create an adverse environment which may facilitate the development of PEs. Notably, however, the effect sizes observed for the PE-Experienced Only group were much smaller than those observed for the Diagnosed and PE-Impaired groups. Secondly, despite not necessarily experiencing more PEs (whether distressing or not) than the PE-Impaired group, Diagnosed individuals had significantly lower functioning, globally. These greater deficits in functioning may have acted as a catalyst to receiving a diagnosis (Addington et al., 2019; Riecher-Rössler & Studerus, 2017) or may, in part, be the result of antipsychotic medication side effects (Tandon et al., 2020) and the social stigma that accompanies a psychotic disorder diagnosis (Degnan, Berry, Humphrey, & Bucci, 2021).

### PE prevalence and symptom expression

Sixty per cent of the sample endorsed at least one PE; this is high compared to other general population studies (12–28%; Kendler, Gallagher, Abelson, & Kessler, 1996; Nuevo et al., 2012; Pignon *et al*. 2018; van Os, Hanssen, Bijl, & Ravelli, 2000). However, this finding is not necessarily inconsistent with studies which have utilised a schizotypal personality measure; for example, a study of schizotypal personality traits in adolescents reported that 93.6% of the sample endorsed at least one of 22 items (Fonseca-Pedrero, Paíno-Piñeiro, Lemos-Giráldez, Villazón-García, & Muñiz, 2009). Social/interpersonal experiences were the most likely to be endorsed across all groups, followed by cognitive/perceptual and disorganised PEs. This finding was not unexpected given that firstly, social/interpersonal difficulties are not unique to psychosis symptomology and also social isolation and exclusion have been heavily implicated in theories of psychosis aetiology (e.g. Hoffman, 2007). Furthermore, non-social PEs that were most commonly reported in the absence of distress/impairment or diagnosis included ‘force’ and ‘strange ideas’; phenomena which may align with spiritual or faith-based experiences and thus may not be considered as distressing.

Despite similar PE expression across groups, the PE-Impaired group were more likely to endorse all PEs individually compared to the PE-Experienced Only group and most PEs compared to the Diagnosed group. They were also more likely to endorse greater numbers of PEs than the PE-Experienced Only group, and unexpectedly, a greater number of impairing and non-impairing PEs compared to the Diagnosed group. Furthermore, only three-quarters of individuals in the Diagnosed group reported ‘lifetime’ endorsement of PEs and only a quarter reported being distressed by one or more PEs. Given that these individuals had been clinically diagnosed with a psychotic disorder/episode, this finding has a number of implications. Firstly, the lower prevalence of most PEs among the Diagnosed group could indicate that, at its most severe, psychosis is characterised by a narrower, more specific phenotypic expression and that only particular PEs are associated with progression to the extreme end of the continuum. The main phenomenologically distinguishing feature associated with the upper end of the continuum (i.e. Diagnosed group) was both endorsement of, and distress associated with, the ‘Shadows’ item. It was the only item for which the Diagnosed group had a significantly greater proportion of experience (7.1% *v.* 5.5%) and distress (3.5% *v.* 2.0%) endorsement compared to the PE-Impaired group. Given that auditory and visual hallucinations are the hallmark symptoms for schizophrenia, experiencing or being distressed by this symptom may be more likely to result in a diagnosis than other symptoms.

A second reason for the apparently lower prevalence of PEs in the Diagnosed group might be the failure of the PE measure to capture negative symptoms. In previous research, measures of schizotypy have tended to correspond to measures of positive schizophrenic symptomology (Cochrane, Petch, & Pickering, 2010; Thomas et al., 2018), but they may be less efficient at measuring negative psychosis symptomology (Cochrane et al., 2010). Thus, individuals in the Diagnosed group may be in a more advanced stage of their disorder, characterised by a greater number of negative symptoms and cognitive impairments than positive symptoms. The differences between the Diagnosed and PE-Impaired groups could also indicate a lack of insight into symptomology in the former, which is common among individuals diagnosed with schizophrenia (Lincoln, Lüllmann, & Rief, 2007), or the impact of treatment (especially antipsychotic medication) which would be expected to reduce the experience of PEs. Finally, the Diagnosed group were the oldest in the sample whereas the PE-Impaired group was the youngest (see online Supplementary Table S1). Typical age of onset of psychosis is late teens or early adulthood (Kessler et al., 2007) but patients often have a long history of diagnosed psychosis (Perkins, Gu, Boteva, & Lieberman, 2005) and the probability of being diagnosed and treated presumably increases with age. Hence, some in the PE-Impaired group may be individuals who, in the future, may enter the Diagnosed group.

### Predictors of group membership

As expected, the predictor variables operated similarly across the continuum, with risk varying in a quantitative rather than a qualitative way. The trauma variables, diagnostic variables and other drug use variables, in particular, differentiated between all levels of the continuum, with the exception of the extreme end (PE-Impaired *v.* Diagnosed). Only a few variables distinguished the Diagnosed from the PE-Impaired group at the upper end of the continuum: female sex, older age, unemployment, parental mental health hospitalisation and lower likelihood of having experienced physical assault. The specific link with parental mental health hospitalisation during childhood could be suggestive of a genetic component to experiencing psychosis at a clinical level; however, there may also be a bias toward diagnosing individuals who have a known family history of psychosis. Moreover, most individuals are unemployed in the month prior to first-episode psychosis (Ramsay, Stewart, & Compton, 2012) and many continue to be unemployed after diagnosis (Marwaha & Johnson, 2004).

A lower likelihood of physical assault may seem inconsistent with the extant evidence base (e.g. Shevlin, Houston, Dorahy, & Adamson, 2008; Varese et al., 2012); however, the comparison here was between PE experienced with impairment and clinical psychosis. It is notable that those classified as PE with impairment were over one and half times more likely to experience physical assault compared to those classified as PE experience only. It may be the case that diagnosis and associated clinical intervention affords some level of protection from social adversities that have been commonly associated with psychosis in the general population.

While the strength of ORs generally indicated that the predictor variables were more strongly associated with the PE-Impaired over the PE-Experienced Only group, in contrast to the hypotheses, this trend could not clearly be extended to the Diagnosed group. For example, other drug use since last interview, past year MDD and GAD, and sexual and physical assault were more strongly associated with the PE-Impaired than Diagnosed group. As suggested above, this finding could reflect the treatment and support the Diagnosed group may have access to, improving their mood, reducing their recent substance use and offering some protection from adversity.

### Limitations

There are several limitations which must be acknowledged. Firstly, the high proportion of PE items endorsed, with and without distress/impairment in the PE-Experienced Only and PE-Impaired group is, in part, a product of the method of categorisation, i.e. that individuals must have endorsed at least one PE to qualify for either group and must have been impaired/distressed by at least one PE to qualify for the PE-Impaired group. Secondly, diagnosed status was self-report and not based on a clinical measure. This group included individuals who may have experienced a single psychotic episode alongside those with a more extensive history of psychosis, therefore heterogeneity within groups is likely.

Thirdly, in the current study, PEs were derived from a schizotypal personality measure. While this measure was a trait-based assessment it still captured experiential accounts pertaining to both thoughts and perceptions. Moreover, use of a schizotypal personality scale as a proxy for experiential assessment is consistent with many other studies. For example, in a recent systematic review on definitions and assessments of psychotic-like experiences (PLEs), Lee et al. (2016) showed that a significant proportion of reviewed studies used schizotypal personality measures to investigate PLEs. Furthermore, studies have shown that measures of schizotypal personality provide non-clinical analogues of the heterogeneous symptomatology found in schizophrenia, although, as previously mentioned, they may be more aligned to positive rather than negative psychosis symptomology (Cochrane et al., 2010). Importantly, a distinction must be acknowledged between the assessment of schizotypal personality, which are usually considered as stable traits across time and other measures of PEs, which adopt a symptom or state approach (Pedrero & Debbané, 2017). Finally, predictor variables were dichotomised for the purpose of the analysis and as such, they tended to capture the presence, rather than severity/frequency of the events (e.g. trauma, drug use).

## Conclusion

The current findings have advanced our understanding of the psychosis continuum in three important ways. First, they have shown that impaired functioning is associated with psychosis experience at all levels of the phenotype and that it is not specifically reflective of distressing and clinically captured experiences only. Second, they have shown that symptom expression is variable along the continuum, becoming most pronounced among those experiencing distressing PEs but without a psychosis diagnosis. Third, commonly recognised and evidenced risk factors for psychosis generally operate consistently along the continuum in a graded, incremental way, with a few notable exceptions that may be informative for clinical assessment (e.g. family history of psychosis). While these findings will need to be replicated, the proposed questions and analytic framework highlight the importance of continuum-based interpretations of the psychosis phenotype and afford valuable opportunities to consider how and in what way/context individuals transition from one position to the next.

## Supporting information

Butter et al. supplementary material 1 Butter et al. supplementary material

Butter et al. supplementary material 2 Butter et al. supplementary material

## Supplementary material

The supplementary material for this article can be found at https://doi.org/10.1017/S0033291723001046
