# Supplementary material for: Functioning, symptom expression and risk along the psychosis continuum
Source: Psychol Med. 2023 Apr 24;53(15):7407–17. doi: 10.1017/S0033291723001046 (PMC10719677; doi:10.1017/S0033291723001046)
Supplement: Butter et al. supplementary material 1 — Butter et al. supplementary material [file S0033291723001046sup001.docx]

**Supplementary Material**

Online supplementary material for ‘Functioning, symptom expression and risk along the psychosis continuum’ (Butter et al., 2023) submitted to *Psychological Medicine*. Corresponding author: Dr Sarah Butter, [s.butter@ulster.ac.uk](mailto:s.butter@ulster.ac.uk)

**Supplementary Table S1: Sociodemographic characteristics of the sample ^1^**

|  |  |  |  |  |  |
| --- | --- | --- | --- | --- | --- |
|  | No PEs  12,505 (37.6) | PE-Experienced Only  15,893 (45.4) | PE-Impaired  3,119 (8.6) | Diagnosed  1,205 (3.1) | Total  34,653 (100.00) |
| **Sex** |  |  |  |  |  |
| Male | 4,971 (44.8) | 6,902 (50.1) | 1,410 (51.4) | 458 (43.9) | 14,564 (47.9) |
| Female | 7,534 (55.2) | 8,991 (49.9) | 1,709 (48.6) | 747 (56.1) | 20,089 (52.1) |
| **Age** |  |  |  |  |  |
| ≤24 | 743 (7.1) | 1,059 (8.2) | 265 (10.0) | 48 (5.2) | 2,183 (7.6) |
| 25-34 | 2,142 (18.1) | 2,682 (17.7) | 624 (21.9) | 162 (14.0) | 5,846 (17.8) |
| 35-44 | 2,788 (21.6) | 3,382 (19.9) | 755 (23.0) | 204 (17.0) | 7,487 (20.6) |
| 45-54 | 2,327 (18.8) | 3,183 (20.6) | 686 (22.1) | 298 (24.3) | 6,825 (20.0) |
| 55-64 | 1,773 (14.3) | 2,365 (14.7) | 436 (13.2) | 231 (18.3) | 5,135 (14.6) |
| 65+ | 2,732 (20.1) | 3,222 (18.9) | 353 (9.9) | 262 (21.1) | 7,177 (19.3) |
| **Ethnicity** |  |  |  |  |  |
| White | 7,691 (73.2) | 8,965 (69.6) | 1,787 (70.7) | 622 (65.7) | 20,161 (70.9) |
| Other ethnicity | 4,814 (26.8) | 6,928 (30.4) | 1,332 (29.3) | 583 (34.3) | 14,492 (29.1) |
| **Unemployed** |  |  |  |  |  |
| Yes | 814 (5.7) | 1,390 (7.9) | 451 (12.7) | 314 (24.6) | 3,179 (8.1) |
| No | 11,691 (94.3) | 14,503 (92.1) | 2,668 (87.3) | 891 (75.4) | 31,474 (91.9) |
| **Children** |  |  |  |  |  |
| Yes | 9,760 (78.1) | 12,168 (76.4) | 2,246 (72.6) | 912 (75.9) | 26,518 (76.9) |
| No | 2,699 (21.9) | 3,675 (23.6) | 866 (27.4) | 288 (24.1) | 7,935 (23.1) |
| **Education** |  |  |  |  |  |
| Completed high school | 10,513 (86.4) | 13,493 (86.5) | 2,645 (86.5) | 960 (81.1) | 29,139 (86.0) |
| Less than high school | 1,992 (13.6) | 2,400 (13.5) | 474 (13.5) | 245 (18.9) | 5,514 (14.0) |
| **Relationship status** |  |  |  |  |  |
| Married/living with partner | 7,439 (68.4) | 8,565 (62.9) | 1,498 (57.7) | 506 (52.2) | 18,866 (63.8) |
| Not married/living with partner | 5,066 (31.6) | 7,328 (37.1) | 1,621 (42.3) | 699 (47.8) | 15,787 (36.2) |

*Note.* ^1^ Unweighted N, weighted using survey design variables %

**Supplementary Table 2. PE item distress/impairment endorsement across total sample and PE groups ^1^**

| **Item** | **Label** | ***N* (%)** | | | **χ^2^, df, *p*** |
| --- | --- | --- | --- | --- | --- |
|  |  | Total sample  34,653 (100.0) | PE-Impaired  3,119 (8.6) | Diagnosed  1,205 (3.1) |  |
| **Social/Interpersonal** |  |  |  |  |  |
| Have you had trouble expressing your emotions and feelings? | Express | 1762 (5.0) | 1510 (51.3) | 135 (10.9) | 567.46 (1), *p*<0.001 |
| Have you rarely shown emotion? | Emotion | 749 (2.1) | 635 (21.1) | 70 (5.4) | 145.93 (1), *p*<0.001 |
| Often you felt nervous when with other people even whom you have known for a while? | Nervous | 491 (1.3) | 369 (11.7) | 84 (6.4) | 25.65 (1), *p*<0.001 |
| Have you felt suspicious of people, even if you have known them for a while? | Suspicious | 1033 (2.6) | 845 (25.3) | 127 (8.5) | 143.86 (1), *p*<0.001 |
| Have you often had the feeling of being watched or stared at, when around people? | Watched | 682 (1.7) | 520 (15.3) | 112 (9.1) | 27.00 (1), *p*<0.001 |
| Have there been very few people that you’re really close to outside of your immediate family? | Close to | 492 (1.3) | 387 (12.5) | 78 (4.7) | 54.46 (1), *p*<0.001 |
| Any Social/Interpersonal |  | 3094 (8.4) | 2632 (85.5) | 255 (20.2) | 1649.11 (1), *p*<0.001 |
| **Disorganised** |  |  |  |  |  |
| Have people thought you acted strangely? | Act strange | 455 (1.1) | 338 (10.0) | 80 (5.5) | 20.91 (1), *p*<0.001 |
| Have people thought you have strange ideas? | Strange ideas | 524 (1.3) | 406 (12.1) | 76 (5.6) | 38.37 (1), *p*<0.001 |
| Have people thought you are odd, eccentric, or strange? | Odd | 438 (1.1) | 338 (10.5) | 68 (4.9) | 32.29 (1), *p*<0.001 |
| Any Disorganised |  | 883 (2.2) | 682 (20.9) | 128 (9.2) | 78.20 (1), *p*<0.001 |
| **Cognitive/Perceptual** |  |  |  |  |  |
| Have you had personal experiences with the supernatural? | Supernatural | 210 (0.5) | 150 (5.0) | 42 (2.8) | 9.22 (1), *p<*0.001 |
| Have you had the sense that some force is around you, even though you cannot see anyone? | Force | 268 (0.6) | 204 (6.2) | 41 (2.7) | 19.75 (1), *p*<0.001 |
| Have you believed that you have a “sixth sense” that allows you to know and predict things that others can’t? | Sixth | 222 (0.5) | 170 (4.8) | 27 (2.1) | 16.00 (1), *p*<0.001 |
| Have you often seen auras or energy field around people? | Auras | 68 (0.2) | 53 (1.4) | 12 (1.0) | 0.76 (1), *p*>0.05 |
| Have you ever felt you could make things happen just by making a wish or thinking? | Happen | 166 (0.4) | 123 (3.3) | 32 (2.6) | 1.33 (1), *p>*0.05 |
| Have you often had the feeling that things that have no special meaning to most people are really meant to give you a message? | Meaning | 397 (0.9) | 311 (8.6) | 66 (5.3) | 12.49 (1), *p*<0.001 |
| Have you often thought that objects or shadows are really people or animals, or that noises are actually people’s voices? | Shadows | 124 (0.3) | 68 (2.0) | 42 (3.5) | 8.03 (1), *p*<0.05 |
| Any Cognitive/Perceptual |  | 931 (2.2) | 721 (20.1) | 134 (10.6) | 51.91 (1), *p*<0.001 |
| **No. PE items** |  |  |  |  |  |
| 0 |  | 30,971 (90.1) | 0 (0.0)^a^ | 905 (76.6)^b^ | 3105.84 (3.68), *p*<0.001 |
| 1 |  | 1949 (5.3) | 1722 (56.1)^a^ | 88 (7.6)^b^ |  |
| 2 |  | 797 (2.1) | 676 (21.8)^a^ | 61 (4.9)^b^ |  |
| 3 |  | 354 (1.0) | 292 (9.8)^a^ | 35 (2.3)^b^ |  |
| 4 |  | 228 (0.6) | 183 (5.2)^a^ | 31 (2.7)^b^ |  |
| 5 or more |  | 354 (0.8) | 246 (7.0)^a^ | 85 (5.9)^b^ |  |

*Note.* ^1^ Unweighted N, weighted using survey design variables %; Chi-squares based on weighted, clustered and stratified data; ^abc^Group comparisons based on weighted only data; Data missing across PE items 0.6 – 1.3%.
